# Honeycomb-shaped electro-neural interface enables cellular-scale pixels in subretinal prosthesis

**DOI:** 10.1038/s41598-019-47082-y

**Published:** 2019-07-23

**Authors:** Thomas Flores, Tiffany Huang, Mohajeet Bhuckory, Elton Ho, Zhijie Chen, Roopa Dalal, Ludwig Galambos, Theodore Kamins, Keith Mathieson, Daniel Palanker

**Affiliations:** 1 Department of Applied Physics, Stanford University, Stanford, CA USA; 2 Hansen Experimental Physics Laboratory, Stanford University, Stanford, CA USA; 3 Department of Electrical Engineering, Stanford University, Stanford, CA USA; 4 Department of Ophthalmology, Stanford University, Stanford, CA USA; 5 Department of Physics, Stanford University, Stanford, CA USA; 6 Institute of Photonics, University of Strathclyde, Glasgow, Scotland UK

**Keywords:** Biomedical engineering, Electrical and electronic engineering, Retina, Neurodegeneration

## Abstract

High-resolution visual prostheses require small, densely packed pixels, but limited penetration depth of the electric field formed by a planar electrode array constrains such miniaturization. We present a novel honeycomb configuration of an electrode array with vertically separated active and return electrodes designed to leverage migration of retinal cells into voids in the subretinal space. Insulating walls surrounding each pixel decouple the field penetration depth from the pixel width by aligning the electric field vertically, enabling a decrease of the pixel size down to cellular dimensions. We demonstrate that inner retinal cells migrate into the 25 μm deep honeycomb wells as narrow as 18 μm, resulting in more than half of these cells residing within the electrode cavities. Immune response to honeycombs is comparable to that with planar arrays. Modeled stimulation threshold current density with honeycombs does not increase substantially with reduced pixel size, unlike quadratic increase with planar arrays. This 3-D electrode configuration may enable functional restoration of central vision with acuity better than 20/100 for millions of patients suffering from age-related macular degeneration.

## Introduction

Electronic approaches to restoration of sight are rapidly advancing^1^, with some systems approved for clinical use in patients blinded by inherited retinal degeneration (retinitis pigmentosa, RP)^2–4^, and a few others in clinical trials. Despite the loss of photoreceptors, the remaining inner retinal neurons survive and can respond to electrical stimulation by eliciting visual percepts^5^. Patients affected by RP and implanted with the epiretinal prosthesis Argus II (Second Sight Medical Products, Inc, Sylmar, CA)^2^ or the subretinal Alpha IMS/AMS (Retina Implant AG, Reutlingen, Germany)^3^ demonstrated improved performance in ambulation and visual search, with the best reported visual acuities of 20/1260 and 20/546, respectively. Although encouraging, those benefits are not sufficient to help with the most prevalent form of retinal degeneration, age-related macular degeneration (AMD), where patients lose high-resolution central vision but retain peripheral field with acuity typically no worse than 20/400.

Visual acuity of 20/200, the limit of legal blindness in the United States, geometrically corresponds to a pixel pitch of about 50 μm^6^. Safe charge injection across the electrode-electrolyte interface limits the minimum size of the electrode. In addition, cross-talk between neighboring electrodes increases with decreasing pixel size^6^. The latter issue can be addressed by providing a circumferential return electrode in each pixel^6^ or utilizing sequential activation with current steering techniques to shape the electric field^7^. Although useful, these approaches further reduce penetration depth of electric field into tissue or limit the number of electrodes available for concurrent stimulation.

The electrode-tissue separation in subretinal space can be reduced using pillar electrodes^8^. Migration of the retinal cells of the inner nuclear layer (INL) to fill the voids in such a 3-D implant brings the target neurons closer to the stimulating electrodes, thereby reducing the stimulation threshold^6,8–10^. However, such pillar electrodes decreased the stimulation threshold only by a factor of two and did not enable continued reduction of the pixel size below 55 μm^8,11^. The fundamental problem limiting the electrode size is the shape of the electric field, which expands from a small electrode and returns to another electrode under the target cells.

**Figure 1:**
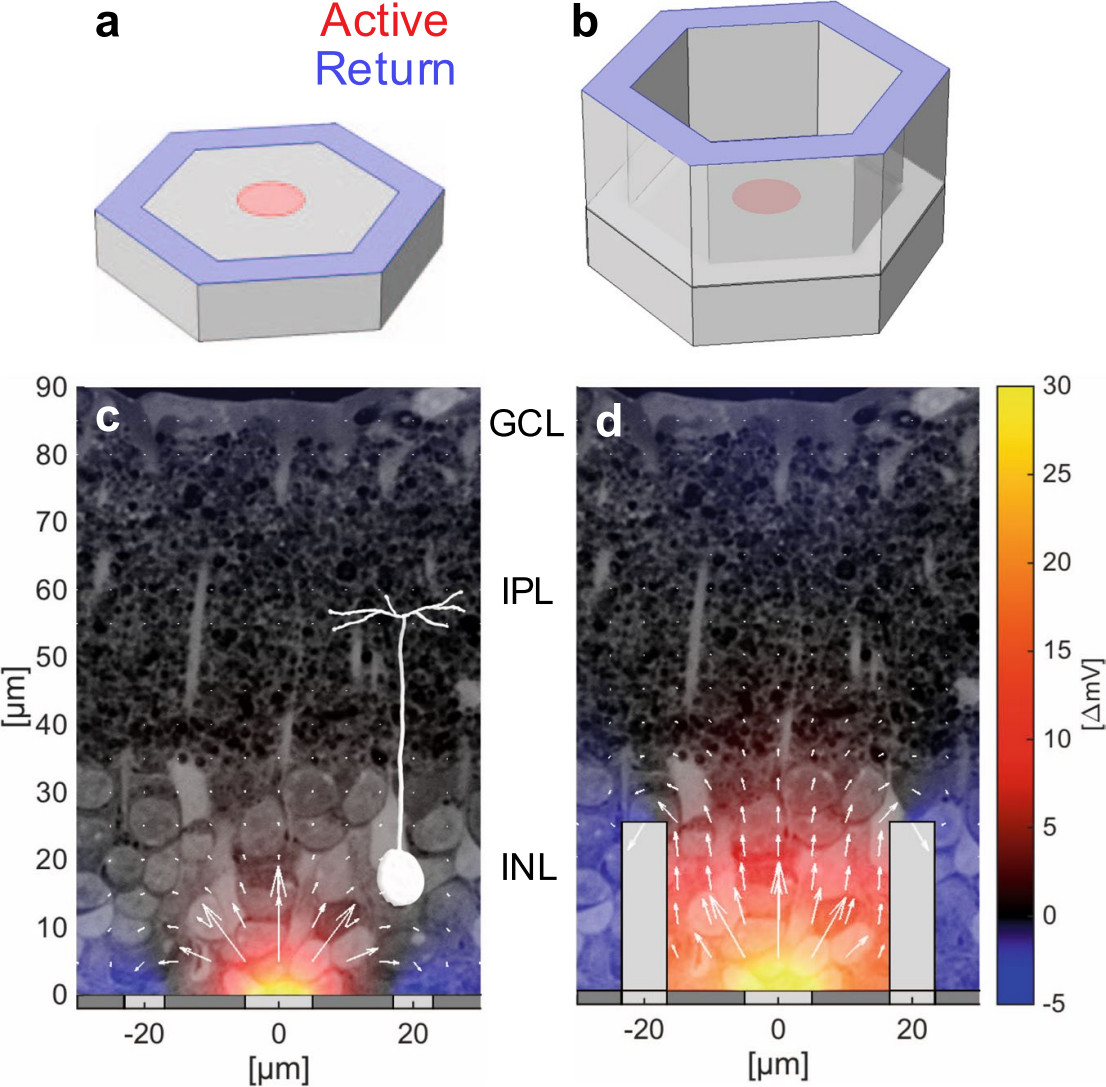
Flat (**a**) and honeycomb (**b**) configurations of the return electrode for a subretinal prosthesis. (**c**) Planar pixels with circumferential returns generate locally confined electric fields with shallow vertical penetration. Electric potential (shown in false color scale for 68 nA current) is represented with respect to potential in the middle of IPL, where axon terminals of bipolar cells reside. (**d**) Return electrodes on top of the insulating walls create a vertical dipole confined to the pixel volume and thereby maximize the vertical potential drop across the target cell layer. Current magnitude (arrow length) is shown in log scale.

Here, we present a novel 3-D geometry for a subretinal prosthesis, which we call the honeycomb configuration, to overcome these limitations and enable scaling the pixels down to cellular dimensions. In this approach, return electrodes are elevated on vertical insulating walls surrounding each pixel (Fig. 1), which align the electric field vertically, matching the orientation of bipolar cells in the retina, and thereby reducing the stimulation threshold. These walls also decouple the field penetration depth from the pixel width, enabling a decrease of the pixel size down to cellular dimensions. We first study the anatomical integration of such 3-D structures with the retina using implants with 20, 30 and 40 µm pixels, and then theoretically quantify the electrical stimulation capabilities using a network-mediated retinal stimulation model validated by experimental measurements. Our results indicate that such technology opens the door to prosthetic vision with acuity better than 20/100, which would be highly beneficial not only for patients completely blinded by RP, but also for restoration of central vision in the much larger population of patients with AMD.

## Results

### Anatomical integration

**Figure 2:**
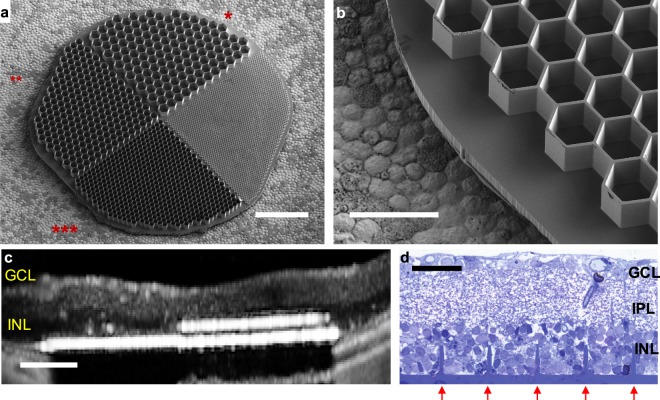
Subretinal honeycomb implants. (**a**) A 1 mm wide device with 25 µm deep honeycombs of 40(*), 30(**), and 20 µm(***) pixel pitch. The fourth quadrant (“flat”) contained a 10 µm pitch structure, which was beyond the processing limit and hence did not develop. (**b**) Higher magnification of the honeycombs with 30 µm pitch. (**c**) OCT image of the subretinal implant in RCS rat 6 weeks post-op. INL (dark layer) is no longer present in front of the 40 µm cavities (right) but is still visible above the “flat” region (left) and outside the implant. (**d**) Histology of the RCS retina with implanted 40 µm honeycomb array. Retinal layers appear preserved, while the INL cells filled the wells (etched and refilled walls indicated by red arrows). Scale bars 200 µm (**a**), 50 µm (**b**), 100 µm (**c**), and 40 µm (**d**).

To assess integration of the honeycombs with the degenerate retina, silicon arrays of 1 mm in diameter were implanted into the subretinal space of RCS rats (P180-300, n = 6) for 6 weeks. Each array was divided into quadrants containing honeycombs with 40, 30, and 20 μm pitch and a flat area (Fig. 2a). Arrays were fabricated in silicon using a Bosch etching process to define the 25 μm deep honeycomb chambers (Fig. 2b). Implant integration with the retina was monitored *in-vivo* by optical coherence tomography (OCT) (Fig. 2c). Six weeks after implantation, the INL is barely detectable by OCT above the honeycombs (Fig. 2c, right) but visible above the flat quadrant (Fig. 2c, left) and outside the implant, indicating INL migration into the cavities.

Histology confirmed migration of the INL cells, showing no visible signs of fibrosis or trauma (Fig. 2d). The retinal structure remains preserved, with clearly defined INL, inner plexiform layer (IPL), and ganglion cell layer (GCL). Although some of the INL cells remain above the honeycomb walls, cavities are completely filled with the densely packed cells down to the base of the array.

**Figure 3:**
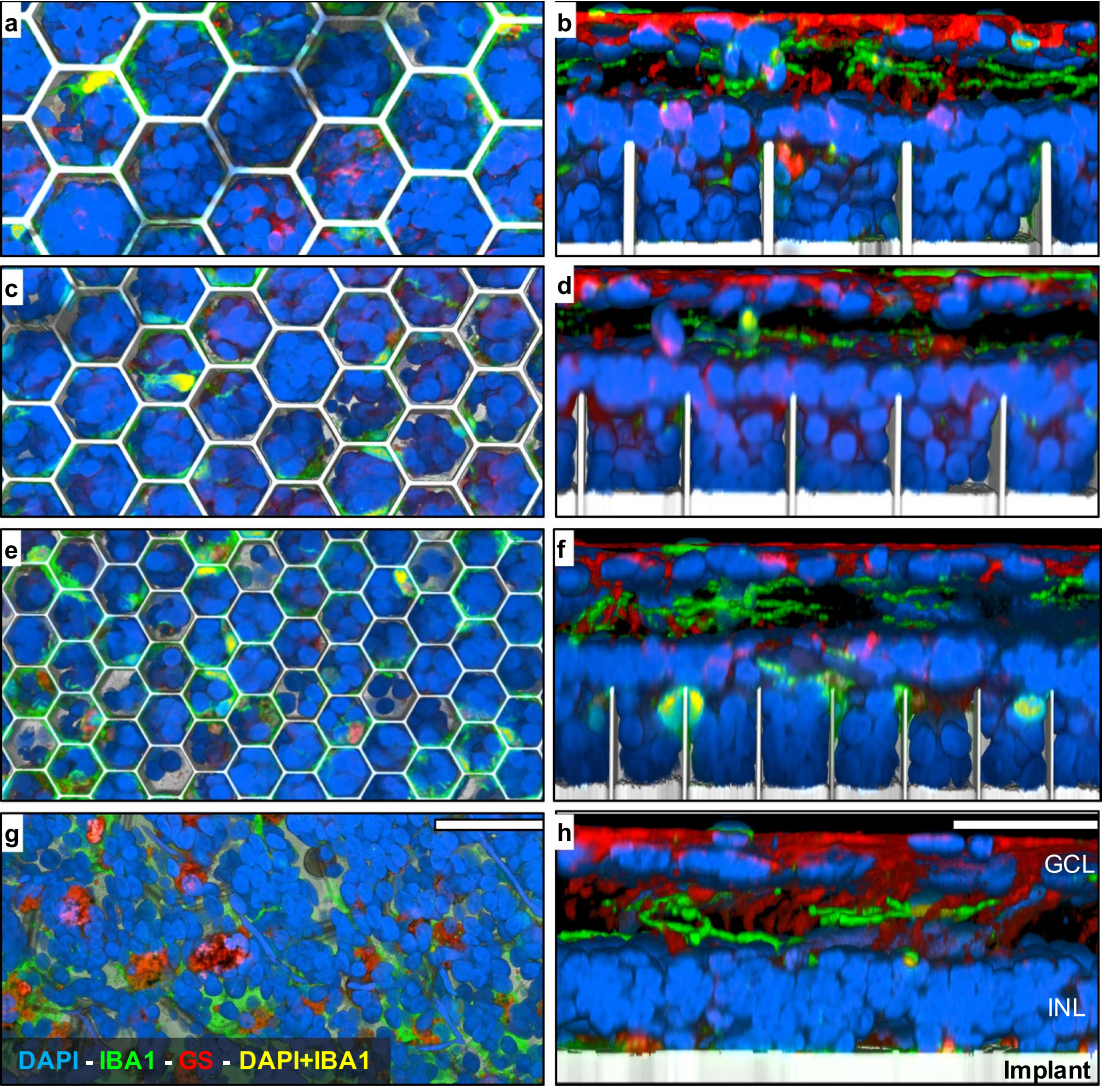
3-D reconstruction from confocal microscopy of the retina with honeycomb structures of 40 µm (**a**,**b**), 30 µm (**c**,**d**) and 20 µm (**e**,**f**) pitch, as well as a flat implant for control (**g**,**h**). Color code: cell nuclei (DAPI, blue), microglia (IBA1, green), Müller glia (GS, red), microglia nuclei (DAPI + IBA1, yellow), implant (reflected light, gray). Left column shows top-down view from the upper surface of the honeycomb. Right column shows cross-section through a row of the honeycomb cavities. INL nuclei densely pack honeycomb cavities of all sizes and migrate down to the base. Co-localization of DAPI with IBA1 illustrates minimal persistent microglia infiltration within the cavities. Scale bars 40 µm.

**Figure 4:**
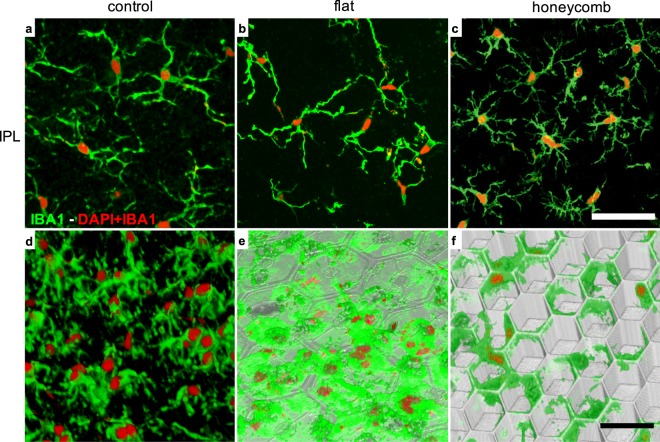
Microglia in the RCS retina. (**a**,**d**) without a device, (**b**,**e**) with a flat implant and (**c**,**f**) with a 20 µm honeycomb array. (**a**–**c**) Maximum projection of a z-stack through the inner plexiform layer (IPL) showing the microglia processes. In all 3 settings microglia exhibit extended processes, indicating the resting state. (**d**–**f**) 3-D reconstruction below the INL. (**d**) Microglia processes and cell bodies in the intact RCS retina extend below the INL. (**e**) These processes spread along the flat implant surface, and hence appear wider than in the intact RCS retina. With the honeycomb implant, microglia processes appear mostly at the upper plane of the array, with minimal extension into the wells. Scale bars 50 µm (**a**–**c**) and 30 µm (**d**–**f**).

**Figure 5:**
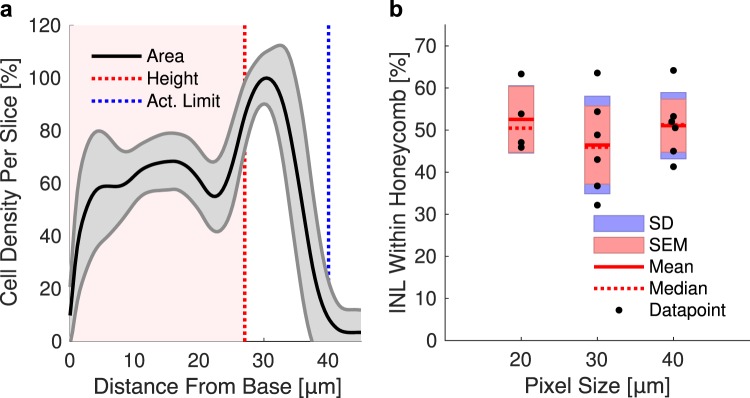
(**a**) Cell density relative to the maximum density in the stack, as a function of distance from the cavity base, for 40 µm honeycombs on a single implant. Solid lines and shaded regions represent the mean and standard deviation of all analyzed cavities, respectively. Extension of electric field to 40 µm within the safe charge injection limit (dashed blue line) enables activation of cells located above the wells. **b**) Fraction of the INL cells within the honeycombs of 40, 30, and 20 µm in width represents 51, 46 and 53% of the total INL volume, respectively.

Assessment of the retinal integration and immune response was performed using 3-D confocal imaging of the whole-mount retina with the implant (Figs 3–4) and subsequent quantification (Fig. 5). 3-D reconstruction from the top of the honeycomb to the base of the cavity (25 μm) reveals dense packing of the INL (DAPI) in most of the observed honeycomb cavities (Fig. 3). Side views through single honeycomb rows, projected from the middle of a cavity to the sidewall, demonstrate migration down to the base of the arrays (Fig. 3, right column). The bottom row in Fig. 3 illustrates the retina with a planar implant, for comparison.

Within the degenerate RCS control retina, extended microglial processes within the IPL indicate the microglial resting state^12–15^ (Fig. 4a), while microglia below the INL extend their processes through the degenerate outer plexiform layer (OPL) (Fig. 4d). With the subretinal implants, microglia in the IPL appear similar to those in the control retina, with extended processes indicating the microglial resting state (Fig. 4b,c). With planar active arrays, microglia reside close to the device surface (Fig. 3, bottom row and Fig. 4b,e). Presence of the cortical response with these active implants^16^ indicates that microglia on a subretinal prosthesis does not prevent electrical stimulation. With the honeycomb implant, microglia processes extend primarily along the top of the walls, with minimal extension into the wells (Fig. 4f).

The extent of retinal integration was assessed by analyzing cell density as a function of height from the base in the cavities of each size (Fig. 5a). An average of 51%, 46% and 53% of the INL cells were found inside the cavities (see Methods) with honeycomb pitch of 40, 30 and 20 μm, respectively (Fig. 5b).

### Modeling retinal response *in-vivo*

To assess the benefits of the honeycomb-shaped arrays, we used a model of network-mediated retinal stimulation. To validate this model, we first compared the modeling results with the *in-vivo* stimulation thresholds measured in rats having planar subretinal photovoltaic implants with various pixel sizes^11,16,17^, and then computed the stimulation thresholds for honeycombs of various sizes.

**Figure 6:**
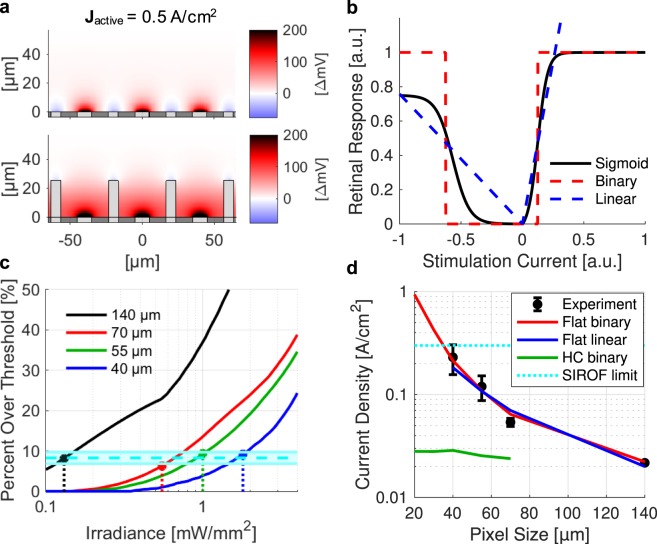
(**a**) Electric potential for planar (top) and honeycomb (bottom) arrays with respect to middle of IPL (z = 57 µm). (**b)** Sigmoidal retinal response (black) can be approximated as a pair of step functions (“binary”, blue) or gradual increase with stimulus (“linear”, red). (**c)** Fitting the binary model to experimental VEP thresholds (stems) indicates that 8.27% ± 1.42% of INL volume in each pixel (cyan) should be above the stimulation threshold to elicit cortical response. (**d)** Experimental and calculated thresholds in terms of current density on active electrode. Both binary (red) and linear (blue) models reproduce the trend observed in experimental measurements with planar devices (black). Honeycomb arrays significantly reduce the stimulation threshold (green), enabling operation below the SIROF charge injection limit (cyan) with pixels smaller than 40 µm.

Complete modeling of this system would require converting the simulated electric field produced by the device (Fig. 6a) into the response of the inner retinal neurons^18^, applying subsequent network-mediated processing to RGC activity, and finally translating the retinal output into the cortical visually evoked potentials (VEP) – an immensely complex modeling task with multiple unknowns. This task can be simplified with the following assumptions: (1) network-mediated stimulation elicits RGC activity that follows a sigmoidal curve^19–21^ and (2) cortical response is driven by summation of the retinal signals and also follows a sigmoidal curve^16,22,23^. We approximated the sigmoidal dependence of the network-mediated retinal response on electric field from previous experiments^16,19^ (Fig. 6b, black) by two extremes: (1) a step function modeling a binary transition across the stimulation threshold (Fig. 6b, red) and (2) a linear function modeling a gradual increase in the neural output in response to increasing stimulus (Fig. 6b, blue). Consequently, the total retinal response is calculated by integrating the cellular responses over the volume of INL with either (1) a binary coefficient, i.e. calculating just the fraction of the INL volume above the stimulation threshold, or (2) with cellular response proportional to its polarization. We will therefore refer to the two models as “binary” and “linear”, respectively.

Electric field in the retina was calculated using a finite element model of the entire arrays in COMSOL Multiphysics 5.0, using the electrostatics module to solve Maxwell’s equations for electric potential, assuming steady-state electric currents. Computed fields were then converted into the retinal response using both the binary and linear models. Each model had only one fitting parameter for relating its retinal output to amplitude of the cortical response. For a binary model, it was the fraction of the INL cells which should be activated to elicit a cortical response, while for a linear model it was the slope of the linear fit. Irradiance is converted into current density based on the pixel geometry and light-to-current conversion efficiency of our 2-diode pixels. To fit the binary model, we therefore compute the electric field vs irradiance and calculate the percent of the INL above the stimulation threshold (4.8 mV for 10 ms anodic pulse, see Methods) for all pixel sizes (Fig. 6c). Using our previously recorded experimental thresholds for 140, 70, 55, and 40 μm pixels (Fig. 6c, stems), we see that to elicit VEP response, 8.27% ± 1.42% of the INL volume should be above the stimulation threshold. Both binary and linear models yield a very similar scaling of the stimulation threshold with the pixel size (Fig. 6d) indicating that the critically important factor is the shape of the electric field, rather than the details of the sigmoidal response curve.

Planar arrays with circumferential returns suffer from a rapid decline of the electric potential along the vertical axis due to (a) the radial spread of electric field from the active electrode and (b) the coplanar return electrode (Fig. 1c). As a result, smaller planar pixels require higher current density to elicit retinal response (Fig. 6d). At some size, required charge density exceeds the charge injection capabilities of the electrode material. For example, for sputtered iridium oxide film (SIROF) commonly used in neurostimulators the limits range from 1 to 10 mC/cm^2^, depending on electrode thickness, pulsing conditions, electrolyte composition, and many other factors^20–23^. Assuming a limit in the middle of this range (3 mC/cm^2^), we see that 10 ms stimulation with pixels smaller than 40 μm requires current density exceeding this charge injection limit (>30 mA/cm^2^).

### Stimulation thresholds with honeycomb arrays

Using the binary model parameters verified by comparison with the *in-vivo* stimulation thresholds obtained with planar implants, we calculated the stimulation thresholds for honeycomb arrays (see details in Methods). These thresholds, in terms of the current density on the active electrode for a 10 ms pulse, are significantly lower than with planar pixels of the same size, and do not increase much with decreasing pixel size (Fig. 6d). Placement of the return electrode on top of the insulating honeycomb walls surrounding the pixel forces the current to flow primarily upward from the active electrode (Fig. 1d), thereby greatly increasing the depth at which the electric potential exceeds the stimulation threshold. In addition, with honeycomb arrays, penetration depth of the electric field into tissue is set by the height of the walls, and hence is decoupled from the pixel width. Therefore, the stimulation threshold in terms of current density does not depend on the pixel width (Fig. 6d), enabling scaling the pixels down to the size limited only by the retinal migration, i.e. by cellular dimensions. Improvements in the stimulation threshold are summarized in Table 1.

**Table 1:** Computed stimulation threshold current densities for different pixel sizes.

Pixel Size (μm)	Planar Threshold (A/cm^2^)	Honeycomb Threshold (A/cm^2^)	Threshold Reduction Factor (Planar/Honeycomb)
70	0.064	0.024	2.70
55	0.11	0.025	4.28
40	0.21	0.028	7.44
30	0.43*	0.028	15.2
20	0.94*	0.028	33.3

Asterisks indicate current densities for a 10 ms pulse which exceed the SIROF charge injection limit.

**Figure 7:**
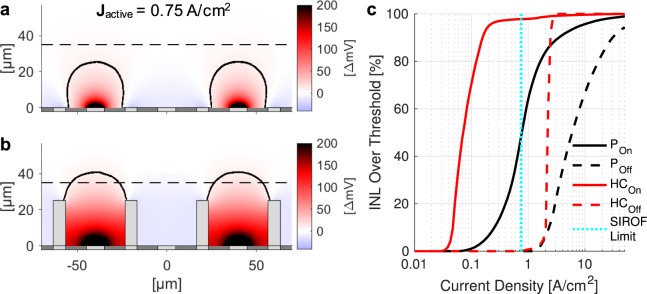
Field potential for activation the planar (**a**) and honeycomb (**b**) arrays with 40 µm pixels using 1-pixel wide stripes of a grating pattern. Black contours outline areas exceeding the stimulation threshold. Dashed line indicates the top of INL. (**c)** Fraction of the INL volume above stimulation threshold vs current density on active electrode. Solid and dashed lines represent ON and OFF pixels, respectively. Honeycombs (red) reduce stimulation threshold and preserve 100% spatial contrast over a wider range, compared to planar arrays (black). The SIROF safe charge injection limit (0.75 A/cm^2^ with 4 ms pulse) is shown with a dashed cyan line.

In addition to stimulation thresholds, honeycomb electrodes significantly improve the spatial selectivity of electrical stimulation (i.e. the contrast between adjacent pixels), which is essential for high-acuity vision. To replicate the grating patterns used for *in-vivo* assessment of visual acuity^16^, we simulate the electric field distribution from our arrays with alternating rows of ON and OFF pixels (Fig. 7a,b). For electrodes of both configurations, increasing current density leads to an increase in positive potential above the ON pixels, as well as negative potential above the OFF pixels. Insulating walls of the honeycomb prevent the lateral spread of the electric field and thereby widen the dynamic range of selective activation of the ON pixels, as shown in Fig. 7c. Further, electric field extends above the walls of the honeycomb to stimulate cells up to 40 µm from the base of the cavity within the safe charge injection limits, allowing for up to 99% activation of the inner retina (Fig. 5a, blue vertical line) without crosstalk.

## Discussion

Our study provides a path toward improvement in spatial resolution of retinal prostheses far beyond the limits of the current systems. Until recently, the best acuity of prosthetic vision achieved in clinical testing was 20/546, observed in 2 patients with the Alpha IMS/AMS subretinal implants^3,24^. With 70 μm pixels, this prosthesis is underperforming by a factor of 2 with respect to its sampling limit, leading some to conclude that continued reduction in pixel size will not improve visual acuity. However, the bottleneck for these devices may be electrical rather than biological: the monopolar configuration of this implant, with active electrodes in each pixel sharing a common remote return electrode, results in a strong cross-talk between neighboring electrodes, greatly reducing spatial contrast^25^. An alternative design that provides active and return electrodes in each pixel, as in our prosthesis^16^, improves the localization of electric field and associated spatial resolution by minimizing electrode cross-talk^26,27^. Indeed, a recent clinical trial with photovoltaic subretinal implants having 100 μm pixels with local return electrodes demonstrated visual acuity up to 20/460 - only 15% below the sampling limit with this pixel size (20/400)^28^. Moreover, in rats, similar implants with 70 μm and 55 μm pixels provided grating acuity also matching the sampling limit^11,16^. This demonstrates that visual acuity can reach the sampling density limit of the stimulating array if it properly confines the electric field in each pixel. However, further decrease in the pixel size is limited by the rapid increase of the stimulation threshold, which rises close to the safety limit with flat pixels of 40 μm width (Fig. 6d).

The radically different geometry of the stimulating array described in this study - the honeycomb configuration - addresses this fundamental limitation. Current from the active electrode at the bottom of the cavity flows upwards due to confinement of the pixel by insulating walls. This simple change in the shape of the electric field dramatically reduces the stimulation threshold. Moreover, one-directional flow of electric current radically changes the scaling of the stimulation threshold with the pixel size. In spherical geometry, an electrode smaller than the distance to target cells can be approximated as a point source. Since the required current does not change with the electrode size in this case, the current density increases inversely proportional to the electrode area, i.e. quadratic with its radius. If the ratio of the electrode size to pixel width is maintained, smaller pixels will require higher current densities, which are limited by the material properties. With one-dimensional flow of current, however, the situation is different: the current density required for a certain potential drop does not change with the pixel width, and therefore pixels can be made much smaller while retaining the same current density on the electrodes. For photovoltaic pixels, it means that the threshold irradiance should remain nearly the same for all pixel sizes, as long as the relative dimensions of electrodes to pixel width do not change.

The honeycomb design is uniquely suited for subretinal placement due to the ability of the inner retinal neurons to migrate into voids in the subretinal space^9,10,29^. Our study demonstrates that the inner retinal neurons readily migrate into wells as small as 18 μm in width (20 μm pixel pitch). Tissue viability after 6 weeks demonstrates that diffusion of oxygen and nutrients from the retinal vasculature located above the implant is sufficient for the cell survival within 25 μm high walls. Since no lower bound of integration was observed in our study, pixel width may continue to scale down, but certainly not below the cell size of about 10 μm. Determination of the exact minimum within this range will require further experimentation. Even without any further decrease in pixel size, arrays with 20 μm pixels should enable spatial resolution matching the natural acuity in rats^16^, and acuity better than 20/100 in humans.

Migration of the majority of the INL into the cavities does not seem to affect the rest of the retinal structure, as cross sections exhibit clearly delineated INL, IPL, and GCL. Since all the connections of the INL cells to RGCs are located above the honeycomb walls in the IPL, we expect that the retinal signal processing will not be affected by this migration. Lateral connectivity between bipolar cells via horizontal cells connected to terminals of photoreceptors in the outer plexiform layer (OPL) is likely missing in the degenerate retina due to absence of photoreceptors. The number of microglia at the honeycomb walls was found to be similar to that with planar devices, and since the latter elicit VEP throughout the life of the animals^16^, immune response with both implants appears to be acceptable. Interestingly, with honeycombs, microglia are localized to the upper portion of the walls, allowing neurons to migrate closer to the stimulating electrode.

Existing subretinal prostheses with a distant return electrode^24^ can also incorporate the vertical walls around pixels. In such a monopolar configuration, polarization of bipolar cells migrating into the wells should become more efficient due to vertical alignment of electric field, and hence the stimulation thresholds as well as the cross-talk between neighboring pixels should decrease. However, without local returns, electric potential inside the dark pixels will still be affected by its illuminated neighbors, and so the dynamic range of the patterns presented on such array will be lower than with the bipolar electrode configuration.

For subretinal implants with photosensitive pixels^19,30^, it is important to keep in mind that the eyeball is shaped such that light incidence on the central retina is vertical, so that the vertically-aligned photoreceptors face the pupil^31^. Therefore, vertical walls of the honeycombs on subretinal implant should not diminish light collection by shadowing the bottom of the wells.

We are now embarking on a major project of designing and testing various processes for fabrication of the active photovoltaic arrays with small pixels and honeycombs – processes facing multiple challenges due to high aspect ratio of various elements in these structures.

## Methods

### Passive honeycomb implants

Passive honeycomb implants were fabricated from crystalline silicon wafers using two mask layers to generate patterns for deep silicon etching. A hexamethyldisilazane (HMDS) primed wafer was spin-coated with 2 µm of negative photoresist (AZ5214-IR) and processed to define the honeycomb walls. This resist was further treated with UV for 15 min to enhance the selectivity during the subsequent etch. 25 μm deep cavities were formed in the exposed silicon regions using a Bosch etch process. After the honeycomb-defining resist was removed, photoresist (7.5% SPR 220-7, 68% MEK, and 24.5% PGMEA) was spray-coated over the wafer to a thickness of 14 μm and processed to define the releasing trenches around the 1 mm wide arrays. A second Bosch process was applied to create these releasing trenches, after which the photoresist was removed. The wafer was spray-coated with a protective 60 µm thick photoresist, and subsequently underwent backside grinding (Grinding and Dicing Services, Inc., San Jose, CA, USA) from 500 to 50 µm in thickness from the base of the honeycombs. Subsequent etching of the remaining excess silicon in XeF_2_ gas completed the release of the implants. The resulting structures are shown in Fig. 2. Cavities are arranged in a hexagonal honeycomb patterns of 40, 30 and 20 µm pitch, having 25 µm high walls of 4, 3, and 2 µm thicknesses, respectively, on a 10 µm thick base. The fourth quadrant was designed for honeycombs of 10 µm pitch, but these were beyond the processing limit for our lithography system and did not develop. We refer to this region as the “flat” quadrant in the text. A 50 nm thick oxide was grown on the silicon implant’s surface to prevent its dissolution *in-vivo*^32^.

### Animals and surgical procedure

All experimental procedures were approved by the Stanford Administrative Panel on Laboratory Animal Care, and conducted in accordance with the institutional guidelines and conformed to the Statement for the Use of Animals in Ophthalmic and Vision research of the Association for Research in Vision and Ophthalmology (ARVO). Animal care and subsequent implantations were conducted as previously described^8,17^ using rats with retinal degeneration from a Royal College of Surgeons (RCS) colony maintained at the Stanford Animal Facility. N = 6 animals were implanted with honeycomb arrays, with implantations occurring between P180 and P300 to ensure complete degeneration of the photoreceptors. Animals were anesthetized with a mixture of ketamine (75 mg/kg) and xylazine (5 mg/kg) injected intramuscularly. A 1.5 mm incision was made through the sclera and choroid 1.5 mm posterior to the limbus. The retina was lifted with an injection of saline solution, and the implant was inserted into the subretinal space. The conjunctiva was sutured with nylon 10-0, and topical antibiotic (bacitracin/polymyxin B) was applied on the eye postoperatively. Surgical success and retinal reattachment were verified using Optical Coherence Tomography (OCT) (HRA2-Spectralis; Heidelberg Engineering, Heidelberg, Germany). The animals were euthanized 6 weeks after implantation. Additional animals for a control group had flat active implants, and were sacrificed after about 6-months long *in-vivo* studies.

### Whole-mount retinal imaging

Animals were euthanized with an intracardiac injection of Beuthanesia, and eyes were enucleated and rinsed in phosphate buffered saline (PBS, Gibco; Thermo Fisher Scientific, Sunnyvale, CA, USA). Anterior segment and lens were removed, and upon locating the implant under a stereo microscope, the eye cup was cut to a 3 mm × 3 mm square centered around the implant, and fixed in 4% paraformaldehyde (PFA; EMS, PA, USA) for 12 hours at 4 °C. The implant was kept in place to prevent tissue damage or reorganization due to its removal. Samples were permeabilized with 1% Triton X-100 (Sigma-Aldrich, CA, USA) in PBS for 3 hours at room temperature. The samples were put in 10% bovine serum albumin (BSA) blocking buffer for 1 hour at room temperature, followed by a 12 hour incubation at room temperature with two primary antibodies; rabbit anti-IBA1 (1:200; Wako Chemicals, VA, USA) and mouse anti-Glutamine Synthetase (GS, 1:100; Novus Biologicals, CO, USA) in 0.5% Triton X-100, 5% BSA in PBS. Samples were washed for 6 hours at room temperature in 0.1% Triton X-100 in PBS (PBS-T) and incubated for 12 hours at room temperature with 2 secondary antibodies: donkey anti-rabbit Alexa Fluor 488 (1:200; Thermo Fisher Scientific, Sunnyvale, CA, USA) and donkey anti-mouse CY3 (1:200; Jackson ImmunoResearch Inc., PA, USA) and counterstained with 4’, 6-Diamidin-2-phenylindol (DAPI) in PBS. After 6 hours of washing in PBST, the samples were mounted with Vectashield (H-1000; Vector Laboratories, Burlingame, CA, USA).

3-D imaging was performed using a Zeiss LSM 880 Confocal Inverted Microscope with Zeiss ZEN Black software. Image planes were acquired through the total thickness of the retina using a Z-stack, with upper and lower bounds defined at the inner limiting membrane (ILM) and 10 μm below the base of the honeycomb cavities, respectively. Stacks were acquired in the center of each honeycomb quadrant using a 40x oil-immersion objective with acquisition area >225 μm ×225 μm and 360 nm z-step.

### Image analysis

Confocal datasets were analyzed using the FiJi distribution of ImageJ^33^. To analyze the cell density in the wells and above the implant, we first maximized the contrast in the individual XY planes to ensure 0.3% channel saturation to correct for brightness variations at different Z positions in the stack. The XY planes were then de-speckled, and background subtracted. A Gaussian blur filter (σ = 3 pixels, 0.42 µm) was applied to smoothen brightness variations within individual cells. The XY planes were then passed through an edge detection filter, and the final image constructed from the background-subtracted OR combination of the processed and edge detected images. For cell density analysis, the channel threshold was adjusted (default method) to provide a binary representation of the cells. The density of cells in the XY plane, defined as the percent of the area occupied by cells, was then computed, taking into account the area occupied by the honeycomb walls. To account for local variations in retinal histology, each honeycomb unit was independently analyzed, with the cell density normalized to the maximum within the unit stack. The percent of INL contained within the cavities is calculated as

$${ \% }_{INL}=\frac{{\int }_{0}^{{z}^{\text{'}}}D(z)dz}{{\int }_{0}^{{z}^{\text{'}\text{'}}}D(z)dz}\cdot 100$$

where *D(z*) is the relative density of cells per XY plane as a function of height (*z*) from the base (*z* = *0*), *z*′ is the wall height (25 µm), and *z″* is the end of INL, defined as a point where D(z) < 0.02.

Six implanted devices, each containing 40, 30, and 20 µm sized honeycombs, were imaged and analyzed. In 2 devices, the 20 µm sized honeycombs were damaged, and so these were excluded from the analysis.

### Histological preparations

After confocal imaging, samples were rinsed in a buffer and fixed in 1.25% glutaraldehyde solution for 24 hours at room temperature. They were then post-fixed in osmium tetroxide for 2 hours at room temperature and dehydrated in graded alcohol and propylene oxide. Following overnight infiltration in epoxy (without DMP-30) at room temperature (Electron Microscopy Sciences - Araldite-EMbed, RT13940, Mollenhauer’s kit), samples were left in an oven for 36 hours at 70 °C. Epoxy blocks were then trimmed until the silicon implants were exposed. To prevent damage to the sectioning knife and formation of silicon debris from the honeycomb structure, the silicon implants were removed using a XeF_2_ etch (Xactix e-1, 23 °C, 3 Torr). Blocks were then refilled with epoxy and put in a vacuum desiccator for two hours, followed by overnight baking at 70 °C. This refilling of the void left after etching of the implant provided structural support during sectioning. The 700 nm thick sections (cut by Reichart UltracutE) were stained with toluidine blue for light microscopy.

### Modeling the electric field and retinal stimulation

Electric field in the retina was calculated using a 3-D finite element model of the complete array in COMSOL Multiphysics 5.0, using the electrostatics module to solve Maxwell’s equations for electric potential, assuming steady-state electric currents. The modeled arrays are 1 mm in diameter, 30 µm thick, and are composed of hexagonal pixels of various sizes, listed in Table 2, with return electrodes connected into a single mesh.

**Table 2:** Number of pixels and their geometry in the modeled photovoltaic arrays.

Pixel Size [µm]	Number of Pixels	Active/pix [μm^2^]	Return/pix [μm^2^]
140	37	1018	3823
70	157	254	1173
55	250	154	853
40	502	79	407
30	930	44	255
20	2172	20	112

The electric field is calculated in a volume (cube, side = 10 mm) for which the ground (0 potential) is defined at the periphery. The modeled prosthesis functions as a closed system, in which all the current injected from active electrodes is collected on the return electrodes. Boundary conditions on electrode surfaces were defined as having a uniform current density, which corresponds to the steady state^34^.

Two electrode configurations were studied: 1) planar with local returns (Figs 1a and 2) honeycomb with local returns (Fig. 1b). In both configurations, a common return electrode collects the current generated by all active pixels such that the total collected current is the sum of the injected currents on individual active electrodes. Side walls of the honeycombs are non-conductive (Fig. 1b). In our previous work with active devices, we assessed spatial resolution *in-vivo* by projecting grating patterns of various spatial frequencies with 100% contrast^16^. The maximum resolution corresponds to activating alternating rows of pixels (ON or OFF rows). To replicate this configuration in simulations, we compute the electric field distribution using an identical activation scheme, and analyze the field at the center of the array, where the cross-talk between neighboring pixels is highest.

Retinal stimulation thresholds were evaluated using a model of network-mediated activation, as previously described^8^. In this approach, we assumed the network-mediated stimulation threshold being defined by a voltage drop across bipolar cells^35^ (Fig. 1c). In an external electric field, the intracellular medium becomes equipotential within a microsecond, resulting in hyperpolarization and depolarization of the cell membrane near and far from the anode, respectively^36,37^. We assumed that retinal bipolar cells respond to stimulation by opening the Ca ion channels in their axon terminals^18^, and that the response of the retinal ganglion cells (RGCs) is proportional to this output above certain threshold, according to the linear-nonlinear (LN) model of retinal circuits^38,39^. In fact, we verified in the past that with photovoltaic subretinal prosthesis, the RGCs response exhibits the features of LN summation, similar to natural vision^16^. To calibrate the model, we used retinal network-mediated stimulation threshold current densities (*j*) for large electrodes from the literature (“long-latency” responses^40^). The 500 µm electrode diameter in that study^40^ is much greater than the retinal thickness, allowing for a uniform field approximation in assessment of the network-mediated simulation threshold. It’s important to keep in mind that cathodic pulses from the large epiretinal electrode are equivalent to anodic pulses produced by a large subretinal electrode. From the average resistivity of the retina (*ρ*, 1000 Ω·cm^35^), and a mean length of bipolar cells, estimated as the distance from the middle of INL to the middle of IPL (*L*, 37 μm), we then calculated a potential difference threshold (*V*_*th*_) from the soma to axon terminals as following:

$${V}_{th}=j\cdot {\rm{\rho }}\cdot L$$

For a 10 ms pulse, *j* = 12.7 μA/mm^2^, yielding *V*_*th*_ = 4.8 mV from soma to axon terminals with anodic stimulation. The cathodic threshold of −21 mV was calculated based on the network-mediated activation curves measured in rat retinas with anodic and cathodic pulse polarities^16,19^, which was scaled to match the calculated anodic threshold^34^.

It is important to note that our model is based on the stimulation current densities from the literature, while the calculated trans-cellular voltage scales linearly with the retinal resistivity, for which there is no consensus in the literature^41–43^. We use the trans-cellular voltage only as a means for assessing the boundaries of the activation zone in tissue, relative to the stimulation threshold. Any variation in assumed retinal resistivity linearly affects the potential for the same current, including the threshold potential. Therefore, these variations do not affect the boundaries of the activation zone since they are calculated relative to the stimulation threshold.

We approximated the sigmoidal dependence of the network-mediated retinal response^16,19,44^ on increasing electric field from previous experiments by two extremes: (1) a step function modeling a binary transition across the stimulation threshold (Fig. 6b red) and (2) a linear function modeling a gradual increase in the neural output in response to increasing stimulus (Fig. 6b blue). Consequently, the total retinal response is calculated by integrating the cellular responses over the volume of INL with either (1) a binary coefficient, i.e. calculating just the fraction of the INL volume above the stimulation threshold, or (2) with cellular response proportional to its polarization. It is important to keep in mind that high above the stimulation threshold, retinal cells may exhibit non-monotonic response^45,46^, which would deviate from the sigmoidal dependence assumed in the current study.

We assume that VEP response is defined by summation of the retinal signals^47^. To find out the fraction of the INL cells above the 1 mm wide implant that should exceed the trans-cellular voltage threshold in order to elicit VEP, we relate the electrical simulations to light intensity corresponding to the VEP thresholds with our photovoltaic prosthesis^16^. *In-vivo* experimental thresholds are reported as a function of irradiance due to the photovoltaic nature of our device, however the electric field simulations are performed for certain current densities. These irradiance thresholds (*E*_*V*_) are therefore converted into current density at the active electrode (*j*_*e*_*)* from

$${j}_{e}=\frac{{A}_{pd}\cdot {E}_{V}\cdot {R}_{pd}}{{A}_{e}}$$

where *A*_*pd*_ is the diode area for the 2-diode configuration, *R*_*pd*_ is the measured light-to-current conversion efficiencies^48^ (0.40, 0.31, 0.26, and 0.24 A/W for 140, 70, 55, and 40 µm pixels, respectively), and *A*_*e*_ is the active electrode area for each pixel size (Table 2).

Analysis of the computed electric fields using these calculated threshold current densities then indicates that for eliciting VEP response, 9.25%, 9.39%, 6.32%, and 8.10% of the INL volume should be above threshold for 140, 70, 55, and 40 µm pixels, respectively (Fig. 6c). The mean of these values is 8.27% ± 1.42%, which we then use for calculating the stimulation thresholds with other pixel configurations. Very consistent fraction of the INL above the threshold for all 4 pixel sizes obtained in the modeling indicates that the model adequately describes the stimulation settings with various configurations of electric field.

To maintain consistency with the previously reported thresholds with our photovoltaic devices, we plot them as function of irradiance (Fig. 6c). However, for assessment of the generalized future structures with not yet defined photosensitive area *A*_*pd*_ and responsivity *R*_*pd*_, and for non-photovoltaic arrays, we represent stimulation thresholds as a function of current density (Fig. 6d).
